# Longer‐term verbal and visual memory patterns in patients with temporal lobe and genetic generalized epilepsies

**DOI:** 10.1002/epi4.12779

**Published:** 2023-08-10

**Authors:** Kristijonas Puteikis, Peter Wolf, Rūta Mameniškienė

**Affiliations:** ^1^ Faculty of Medicine Vilnius University Vilnius Lithuania; ^2^ Center for Neurology Vilnius University Vilnius Lithuania; ^3^ Danish Epilepsy Center Filadelfia Dianalund Denmark; ^4^ Postgraduation Program of Medical Sciences Santa Catarina Federal University Florianópolis Brazil

**Keywords:** amnesia, forgetting, neuropsychology, recall, seizure

## Abstract

**Objective:**

To compare forgetting patterns between patients with temporal lobe (TLE) and generalized (GGE) epilepsies and to assess whether recall is associated with epileptic activity.

**Methods:**

Thirty‐three patients with TLE (13 left, 17 right, and 3 nonlateralized TLE), 42 patients with GGE, and 57 healthy controls (HCs) were asked to recall words, verbal story material, and the Rey‐Osterrieth complex figure at two delays. Accelerated long‐term forgetting (ALF) was defined by group performance comparable to HCs at 30 min and worse recall than HCs after 4 weeks. ALF was assessed by comparing raw test scores in a two‐way repeated measures analysis of variance (ANOVA) adjusted for the learning capacity.

**Results:**

Compared to HCs, patients with R‐TLE remembered fewer items of the word list after 30 min as well as after 4 weeks. Patients with L‐TLE and GGE had comparable learning‐adjusted performance to HCs at the 30 min delay but scored less after 4 weeks (group by delay interaction *F*(3, 124) = 3.2, *P* = 0.026, $\eta_{p}^{2}$ = 0.07). The epilepsy group (patients with TLE and GGE combined) performed as well as HCs at 30 min but worse after 4 weeks irrespective of experienced seizures during the 4‐week delay or interictal bilateral (TLE) or generalized (GGE) activity before the study. We noted no statistically significant differences between patient and HC verbal story (group by delay interaction *F*(3, 124) = 0.7, *P* = 0.570, $\eta_{p}^{2}$ = 0.02) or complex figure (*F*(3, 124) = 0.8, *P* = 0.488, $\eta_{p}^{2}$ = 0.02) recall.

**Significance:**

Our data support verbal and visual memory impairment in both TLE and GGE with different performances between these groups in the task of word recall. We suggest the presence of ALF in patients with GGE and left TLE after adjusting for learning capacity. We could not confirm the influence of epileptic activity on long‐term forgetting patterns. Future studies are required to better define domain‐specific differences in memory impairment in TLE and GGE.

**Key Points**

- Long‐term memory capacity has rarely been compared between temporal lobe (TLE) and generalized (GGE) epilepsies.
- Our data suggest different word list forgetting patterns in epilepsy groups, with the earlier loss of information in the right TLE.
- Patients with epilepsy performed worse than healthy controls in the visual recall task at delays of 30 min and 4 weeks.
- We did not observe significant differences in forgetting curves of verbal stories among patients with TLE, GGE, and healthy controls.

## 1 INTRODUCTION

Cognitive dysfunction in epilepsy is frequent and poses various challenges – its detection requires comprehensive testing of different cognitive domains while options for evidence‐based rehabilitation remain scarce.^1^, ^2^ Current knowledge of cognitive functioning among people with epilepsy (PWE) predominantly emerged from data of presurgical neuropsychological assessment in temporal lobe epilepsy (TLE).^3^ However, individuals with genetic generalized epilepsies (GGE) have later been shown to perform worse than their healthy counterparts in various cognitive tasks as well.^4^ Within the spectrum of epileptic disorders, TLE and GGE may be perceived to be relatively dissimilar conditions. Thus, their impact on cognitive functioning has often been investigated separately. Results of modified memory testing in the two PWE subgroups suggested that a phenomenon of accelerated long‐term forgetting (ALF) – normal memory function after a short (e.g., 1 h) delay with abnormally increased loss of recall afterward – may be present both in TLE and GGE.^5^ Long‐term memory performance in samples belonging to each of these groups, however, has rarely been directly compared.^6^, ^7^ We, therefore, believe that additional consideration of forgetting patterns in TLE and GGE is needed, especially as the presence of ALF has not been robustly established in adults with GGE.^8^, ^9^ A better understanding of long‐term memory function in these two epilepsy groups may provide future research directions related to the mechanisms that underlie ALF. Therefore, the present article aims to directly compare long‐term memory performance between patients with TLE and GGE as well as to investigate whether it is associated with epileptic activity.

## 2 METHODS

### 2.1 Study context and setting

We present an analysis of data emerging from two PWE samples (one TLE and one GGE) and one healthy control (HC) group who underwent the same memory evaluation composed of tasks of word recall (the Lithuanian equivalent to the Rey Auditory Verbal Learning Test, RAVLT), short verbal story recall (VLS), as well as figure recall (Rey–Osterrieth complex figure test, ROCFT), tested at three time points (RAVLT – 5th learning trial, 30 min, 4 weeks; VLS – immediate recall, 30 min, 4 weeks; ROCFT – copy result, 30 min and 4 weeks). Testing of a longer (4‐week) retention interval was selected as ALF may become more evident over longer delays and they may enhance the ecological validity of standard memory tests.^10^, ^11^ The recruitment and examination procedure as well as individual group data have already been described in detail for both TLE and GGE subgroups.^8^, ^12^

Study participants aged 18–60 years were diagnosed with epilepsy at a tertiary center of Vilnius University Hospital Santaros Klinikos using full neurological examination, including waking electroencephalography (EEG) and brain magnetic resonance imaging (MRI). Patients using other medications than antiseizure medications, having progressive or extensive cerebral lesions, psychiatric comorbidities, or significant sensorimotor deficits were excluded. Participants were assigned either GGE or TLE (subdivided as left (L‐TLE), right (R‐TLE), or bilateral) based on EEG and MRI findings. Additional interictal EEG of 30 min was performed before the neuropsychological evaluation. Patients were not tested in the postictal period (24 h after their last seizure). Data regarding patient age, education, employment, seizure frequency, age of onset and duration of epilepsy, and the number of antiseizure medications (ASMs) used were collected upon enrolment during one of the routine outpatient visits and just before the first neuropsychological evaluation time point.

The group of healthy controls (HCs) was composed of health care workers and their relatives or friends from the same hospital, they were matched for age and sex. Individuals with psychiatric or neurological disorders, or those using any form of medication in the past 2 weeks before testing were excluded.

All study participants underwent the same evaluation that included (1) five trials of learning the RAVLT word list A, one‐time learning of a word list B and free recall at 30 min and 4 weeks, (2) immediate, 30 min and 4‐week recall of the VLS, and (3) copy, 30 min and 4‐week recall of the ROCFT. Participants were not informed that they will need to recall the same material for the follow‐up session after 4 weeks and continued the use of ASMs between visits as usual.

The study had received approval from the Vilnius Regional Biomedical Research Ethics Committee (approval no. 2019/12˗1173˗661), and all participants provided their informed consent.

### 2.2 Statistical analysis

Demographic and clinical characteristics were compared between groups using one‐way ANOVA (normal distribution), Kruskal–Wallis *H* test (non‐normal distribution or ordinal variables), and chi‐squared or Fisher's exact tests (categorical variables).

The presence of ALF in individual patients was defined as performance not lower than one standard deviation below the control group mean after 30 min (*z*‐score ≥ −1.0) and lower than the HC mean after 4 weeks (*z*‐score < −1.0). The concordance between ALF detection in the RAVLT, the VLS, and the ROCFT was calculated by Cochran's *Q*‐test.

Long‐term memory function between groups of TLE and GGE was compared by performing a two‐way repeated measures analysis of variance (ANOVA) with one between‐subject (group) and one within‐subject (delay) factor. First, raw scores of each recall task were compared between patients with R‐TLE, L‐TLE, GGE, and HCs at three time points: the last or initial learning trial (5th learning trial of the RAVLT, immediate recall of the VLS or the copy result of the ROCFT), the delay at 30 min and the delay at 4 weeks. In subsequent analyses, raw scores of each recall task were compared between PWE and HCs at delays of 30 min and 4 weeks (i.e., at two time points after learning or immediate recall) while scores of the very first time point (5th learning trial of the RAVLT, immediate recall of the VLS and the copy result of the ROCFT) were entered as covariates to adjust for differences in initial learning. This adjustment was used to counter the possible effects of initial learning or immediate recall on forgetting curves after the selected delays.^13^ While it remains debatable whether information acquisition capacity influences subsequent forgetting, the adjustment served to dissociate learning abilities from the analysis of memory decay.^14^, ^15^, ^16^ It was regarded as an alternative to matching group learning to a criterion, which has been suggested as a quality standard in ALF trials.^13^ In case of a statistically significant group by delay interaction (*P* < 0.05), post hoc testing for between‐group differences in performance at each time point (30 min and 4 weeks) were investigated using a Bonferroni‐adjusted simple main effects analysis.

The analysis described above was repeated by subdividing the group of PWE based on whether patients experienced any seizures (SZ‐/SZ+) or generalized (primary or evolving to) tonic–clonic seizures (GTCS−/GTCS+) during the 4‐week interval between measurements, or whether they had bilateral (TLE) or generalized (GGE) activity on EEG that was performed in relation to the first time point and with the same ASM regimen as during the study (EEG−/EEG+). The a priori known sample size (n = 132) was deemed sufficient for the analysis (1‐*β* > 0.99 if *α* = 0.05, *f* = 0.25, *ε* = 1.0).

IBM SPSS v26 and MS Excel v16 were used for statistical analysis and visual representation, accordingly.

## 3 RESULTS

### 3.1 General findings

The study sample consisted of 33 patients with TLE (mean age 31.6 ± 9.2 years, 12 [37.5%] male), 42 with GGE (mean age 28.1 ± 8.7 years, 10 [23.8%] male), and 57 healthy controls (mean age 27.6 ± 9.3, 20 [35.1%] male). All PWE but one used antiseizure medication (4 [12.1%] of patients with TLE and 24 [57.1%] with GGE were on monotherapy). During the 4‐week interval between visits, 21 (44.7%) patients with TLE and 26 (55.3%) with GGE reported experiencing at least one seizure, among them 9 (42.9%) and 20 (76.9%), respectively, had a generalized tonic–clonic seizure. Three individuals with GGE (7.1%) and none with TLE were seizure‐free during the 3 months preceding the evaluation. There were 13 (30.2%) and 30 (69.8%) patients with TLE and GGE, respectively, with bilateral or generalized epileptiform discharges on EEG. Within the sample of TLE, 17 patients had R‐TLE (structural etiologies included meningioma [n = 2], focal cortical dysplasia [n = 3], heterotopia [n = 1], hippocampal sclerosis [n = 3], postencephalitic lesions [n = 1], traumatic lesion [n = 1], and 6 patients had no identifiable lesions) and 13 had L‐TLE (structural etiologies included arachnoid cyst [n = 1], cavernoma [n = 1], hippocampal sclerosis [n = 4], and 7 had no identifiable lesions). Three patients with TLE had no lateralizing findings. The characteristics of HCs, patients with GGE, R‐TLE, and L‐TLE are presented in Table 1.

**TABLE 1:** Demographic and clinical characteristics of the study sample (patients with nonlateralized TLE were excluded).

Characteristic	Group				Test result	*P*‐value
	HC (n = 57)	GGE (n = 42)	R‐TLE (n = 17)	L‐TLE (n = 13)		
Sex, M/F, n (%)	20 (35.1%)/37 (64.9%)	10 (23.8%)/ 32(76.2%)	6 (35.3%)/11 (64.7%)	5 (38.5%)/8 (61.5%)	2.042 (FE)	0.570
Age, years	27.6 ± 9.3	28.1 ± 8.7	29.5 ± 7.5	32.8 ± 8.9	*H* (3) = 5.454	0.141
Education, n (%)						
Secondary, completed	15 (26.3%)	19 (45.2%)	10 (58.8%)	5 (38.5%)	7.328 (FE)	0.288
Tertiary, student/dropout	24 (42.1%)	13 (31.0%)	4 (23.5%)	4 (30.8%)		
Tertiary, completed	18 (31.6%)	10 (23.8%)	3 (17.6%)	4 (30.8%)		
Employment, n (%)						
Employed	37 (64.9%)	29 (69.0%)	11(64.7%)	9 (69.2%)	26.375 (FE)	<0.001**
Unemployed	0 (0%)	4 (9.5%)	5 (29.4%)	4 (30.8%)		
Student	20 (35.1%)	9 (21.4%)	1 (5.9%)	0 (0%)		
Age at onset, years		15.8 ± 8.7	12.8 ± 7.2	17.5 ± 12.8	*F*(2, 69) = 1.089	0.342
Epilepsy duration, years		12.1 ± 10.1	16.7 ± 7.9	15.2 ± 11.0	*H* (2) = 2.923	0.232
Seizure frequency (3‐month average)		13.7 ± 30.8	10.5 ± 13.2	8.1 ± 7.9	*H* (2) = 10.501	0.005*
Number of ASMs, n (%)						
One		24 (57.1%)	1 (5.9%)	2 (15.4%)	*H* (2) = 25.475	<0.001**
Two		13 (31.0%)	4 (23.5%)	5 (38.5%)		
Three		4 (9.5%)	7 (41.2%)	4 (30.8%)		
Four		1 (2.4%)	5 (29.4%)	2 (15.4%)		
Any generalized seizures between visits, n (%)		26 (61.9%)	8 (47.1%)	12 (92.3%)	1.019 (FE)	0.672

*Note*: *P < 0.05, **P < 0.001.

Abbreviations: ASM, antiseizure medication; FE, Fisher's exact test; GGE, genetic generalized epilepsy; HC, healthy control; L‐TLE, left temporal lobe epilepsy; R‐TLE, right temporal lobe epilepsy.

### 3.2 General results of neuropsychological testing

**FIGURE 1:**
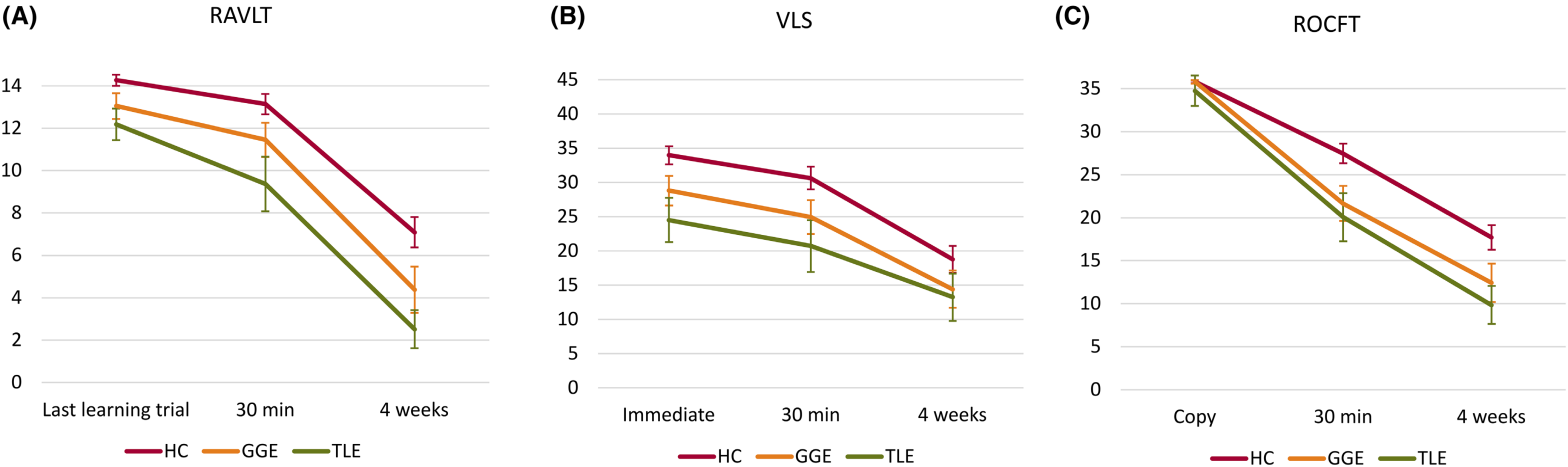
Performance during (A) the Rey Auditory Verbal Learning Test (RAVLT), (B) verbal logical story recall (VLS), and (C) the Rey–Osterrieth Complex Figure Test (ROCFT) between healthy controls (HC) and subgroups of people with epilepsy with either genetic generalized epilepsy (GGE) or temporal lobe epilepsy (TLE). Mean values of test scores and 95% confidence intervals are presented.

Group performance in the RAVLT, VLS task, and ROCFT is presented in Table 2 and Figure 1. In a two‐way repeated measures ANOVA of the RAVLT unadjusted for the last learning trial (group effect *F*(3, 125) = 25.3, *P* < 0.001, $\eta_{p}^{2}$ = 0.38, delay effect *F*(1.7, 212.8) = 615.2, *P* < 0.001, $\eta_{p}^{2}$ = 0.83, group by delay interaction *F*(5.1, 212.8) = 4.5, *P* = 0.001, $\eta_{p}^{2}$ = 0.10), PWE performed worse than HCs at each time point (*P* < 0.05), except for L‐TLE group at the last learning trial (*P* = 0.148). There was no statistically significant group by delay interaction (*F*(4.9, 203.2) = 1.9, *P* = 0.098, $\eta_{p}^{2}$ = 0.04) in a respective analysis of the VLS (group effect *F*(3, 125) = 14.0, *P* < 0.001, $\eta_{p}^{2}$ = 0.25, delay effect *F*(1.6, 203.2) = 160.2, *P* < 0.001). Patients performed as well as HCs in the copy task of the ROCFT (*P* > 0.05), but all PWE subgroups underperformed (*P* < 0.05) after 30 min and 4 weeks (group effect *F*(3, 125) = 16.6, *P* < 0.001, $\eta_{p}^{2}$ = 0.29, delay effect *F*(2, 250) = 666.6, *P* < 0.001, $\eta_{p}^{2}$ = 0.84, group by delay interaction *F*(6, 250) = 7.7, *P* < 0.001, $\eta_{p}^{2}$ = 0.16).

**TABLE 2:** The performance of study groups in the selected memory tasks.

Task	Group				Test result	*P*‐value	
	HC (n = 57)	GGE (n = 42)	L‐TLE (n = 13)	R‐TLE (n = 17)			Post hoc (Bonferroni adjusted)
Verbal memory							
RAVLT‐1st trial	8.5 ± 1.7	7.3 ± 2.4	6.4 ± 1.5	7.5 ± 2.3	*H* (3) = 20.728	<0.001**	HC > R‐TLE HC > GGE
RAVLT‐2nd trial	11.1 ± 2	10.2 ± 2.3	8.6 ± 2.2	9.4 ± 2.5	*H* (3) = 19.321	<0.001**	HC > R‐TLE
RAVLT‐3rd trial	13 ± 1.5	11.8 ± 2.1	9.8 ± 2.2	11.2 ± 2	*H* (3) = 32.563	<0.001**	HC > L‐TLE, R‐TLE, GGE GGE > R‐TLE
RAVLT‐4th trial	13.3 ± 2.2	12.6 ± 1.9	11.2 ± 1.7	12.2 ± 2.4	*H* (3) = 19.395	<0.001**	HC > R‐TLE
RAVLT‐5th trial	14.3 ± 1.0	13.1 ± 1.9	11.5 ± 2.1	13.2 ± 1.6	*H* (3) = 32.402	<0.001**	HC > R‐TLE, GGE GGE > R‐TLE
RAVLT‐B list	8.3 ± 2.0	7.2 ± 2.6	6.2 ± 1.2	6.5 ± 2.4	*H* (3) = 21.951	<0.001**	HC > L‐TLE, R‐TLE, GGE
RAVLT‐30 min	13.1 ± 1.8	11.5 ± 2.6	8.5 ± 3.3	10.7 ± 3.6	*H* (3) = 31.148	<0.001**	HC > R‐TLE, GGE GGE > R‐TLE
RAVLT‐4 weeks	7.1 ± 2.7	4.4 ± 3.5	1.9 ± 2	2.9 ± 2.7	*H* (3) = 47.044	<0.001**	HC > L‐TLE, R‐TLE, GGE
Verbal‐logical memory							
VLS‐immediate	34 ± 4.9	28.8 ± 6.9	22.5 ± 9.1	25 ± 8.5	*F*(3, 125) = 16.912	<0.001**	HC > L‐TLE, R‐TLE, GGE GGE > R‐TLE
VLS‐30 min	30.6 ± 6.3	24.9 ± 8.0	19.1 ± 11.8	20.5 ± 8.9	*F*(3, 125) = 12.858	<0.001**	HC > L‐TLE, R‐TLE, GGE
VLS‐4 weeks	18.7 ± 7.4	14.4 ± 8.7	10.9 ± 9.5	14.6 ± 9.9	*F*(3, 125) = 4.756	0.004	HC > R‐TLE
Visual memory							
ROCFT‐copy	35.8 ± 0.6	35.8 ± 0.7	34.1 ± 6.7	35.3 ± 2.5	*H* (3) = 0.163	0.983	
ROCFT‐30 min	27.5 ± 4.3	21.7 ± 6.6	19.2 ± 9.1	20.4 ± 6.7	*H* (3) = 30.433	<0.001**	HC > L‐TLE, R‐TLE, GGE
ROCFT‐4 weeks	17.7 ± 5.4	12.4 ± 7.2	8.2 ± 6.4	11.2 ± 4.4	*H* (3) = 34.193	<0.001**	HC > L‐TLE, R‐TLE, GGE

*Note*: Mean values of the raw scores and their standard deviations are presented. **P < 0.001.

Abbreviations: GGE, genetic generalized epilepsy; HC, healthy control; L‐TLE, left temporal lobe epilepsy; R‐TLE, right temporal lobe epilepsy; RAVLT, Rey auditory verbal learning test; ROCFT, Rey‐Osterrieth complex figure test; VLS, verbal‐logical story test.

If considered individually, eight (19.0%) patients with GGE, one (5.9%) with R‐TLE (epilepsy etiology – focal cortical dysplasia) and three (23.1%) with L‐TLE (arachnoidal cyst [n = 1], non‐lesional [n = 2]) had had ALF on the RAVLT. The values were, respectively, five (11.9%, GGE), none (R‐TLE) and one (7.7%, L‐TLE, epilepsy etiology – cavernoma) for the VLS task and three (7.1%, GGE), three (17.6%, R‐TLE, focal cortical dysplasia [n = 3]) and two (15.4%, L‐TLE, hippocampal sclerosis [n = 1], non‐lesional [n = 1]) for the ROCFT. According to Cochran's *Q*‐test, the difference in ALF prevalence among individual patients was not statistically significant across the three tasks (*χ* ^2^ (2) = 2.435, *P* = 0.296).

### 3.3 Differences in long‐term memory function in GGE, TLE, and HCs after adjustment for initial learning

**FIGURE 2:**
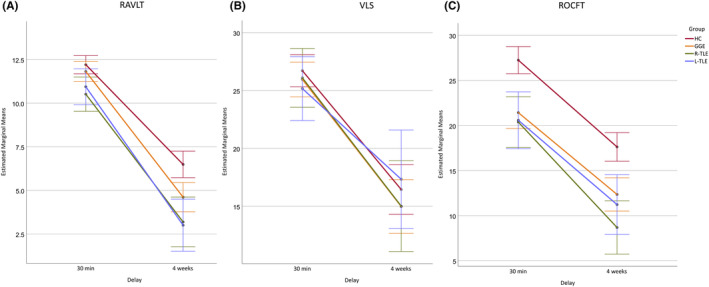
Performance during (A) the Rey Auditory Verbal Learning Test (RAVLT), (B) verbal logical story recall (VLS), and (C) the Rey–Osterrieth Complex Figure Test (ROCFT) at 30 min and 4‐week delays after adjustment for the last learning trial (RAVLT), immediate recall (VLS) or figure copy results (ROCFT). Estimated marginal means and 95% confidence intervals are presented for subgroups of healthy controls (HC) and patients with genetic generalized epilepsy (GGE) as well as right (R‐TLE) or left (L‐TLE) temporal lobe epilepsy.

Comparison of last learning trial‐adjusted results of the word learning task (RAVLT) yielded statistically significant delay by last learning trial interaction (*F*(1, 124) = 5.8, *P* = 0.018, $\eta_{p}^{2}$ = 0.05), group (*F*(3, 124) = 9.1, *P* < 0.001, $\eta_{p}^{2}$ = 0.18) and group by delay interaction (*F*(3, 124) = 3.2, *P* = 0.026, $\eta_{p}^{2}$ = 0.07) effects. Post hoc analysis of the ANOVA revealed that HCs outperformed the R‐TLE group at both testing points (HC > R‐TLE, *P* = 0.031 at 30 min and *P* = 0.001 at 4 weeks) while patients with L‐TLE and GGE performed similarly as HCs at 30 min (HC = L‐TLE, *P* = 0.196, HC = GGE, *P* = 1.000) but worse after 4 weeks (HC > L‐TLE, *P* < 0.001, HC > GGE, *P* = 0.010), Figure 2A.

During the verbal‐logical story task, there were no significant delay (*F*(1, 124) = 0.4, *P* = 0.547, $\eta_{p}^{2}$ < 0.01), group (*F*(3, 124) = 0.4, *P* = 0.779, $\eta_{p}^{2}$ = 0.01) or group by delay (*F*(3, 124) = 0.7, *P* = 0.570, $\eta_{p}^{2}$ = 0.02) interaction effects, suggesting similar performance among groups after adjusting the analysis for immediate recall (Figure 2B). There was a significant delay by immediate recall interaction (*F*(1, 124) = 16.0, *P* < 0.001, $\eta_{p}^{2}$ = 0.11).

Adjusted ANOVA of the ROCFT revealed significant group (*F*(3, 124) = 16.5, *P* < 0.001, $\eta_{p}^{2}$ = 0.29) and delay by ROCFT‐copy interaction (*F*(1, 124) = 6.6, *P* = 0.012, $\eta_{p}^{2}$ = 0.05) effects with no significant delay (*F*(1, 124) = 1.4, *P* = 0.240, $\eta_{p}^{2}$ = 0.01) or group by delay interaction (*F*(3, 124) = 0.8, *P* = 0.488, $\eta_{p}^{2}$ = 0.02) effects, Figure 2C.

### 3.4 Differences in long‐term memory function based on seizure activity

**FIGURE 3:**
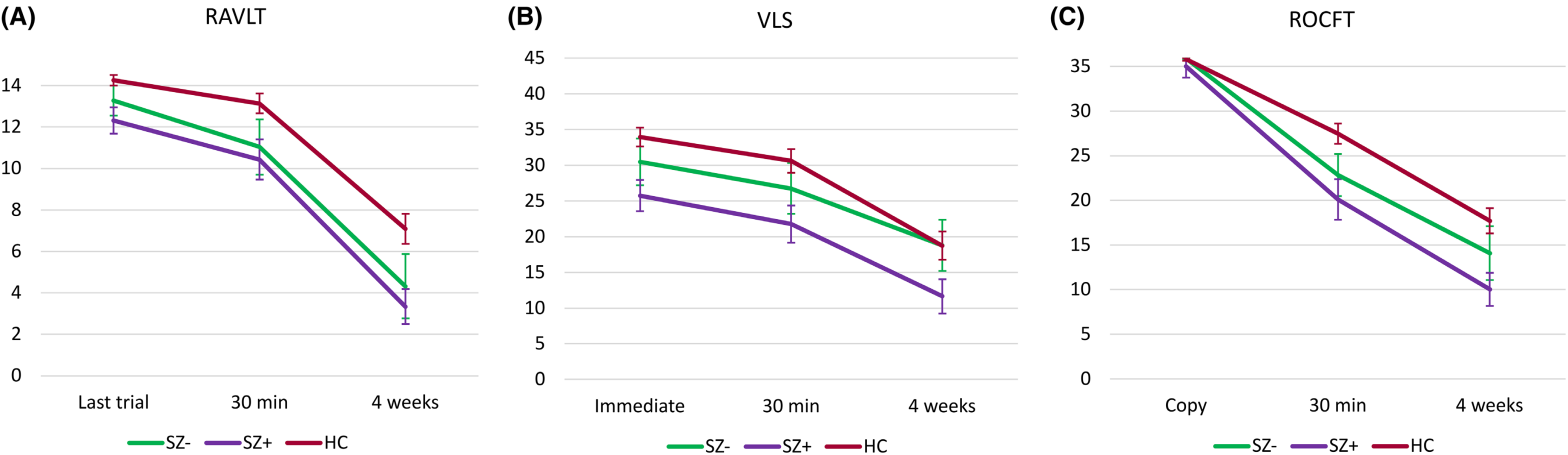
Performance during (A) the Rey Auditory Verbal Learning Test (RAVLT), (B) verbal logical story recall (VLS), and (C) the Rey–Osterrieth Complex Figure Test (ROCFT) in healthy controls (HC) and groups of people with epilepsy subdivided based on any seizures (SZ+/SZ−) experienced within the 4‐week interval between testing occasions. Mean values of test scores and 95% confidence intervals are presented.

**FIGURE 4:**
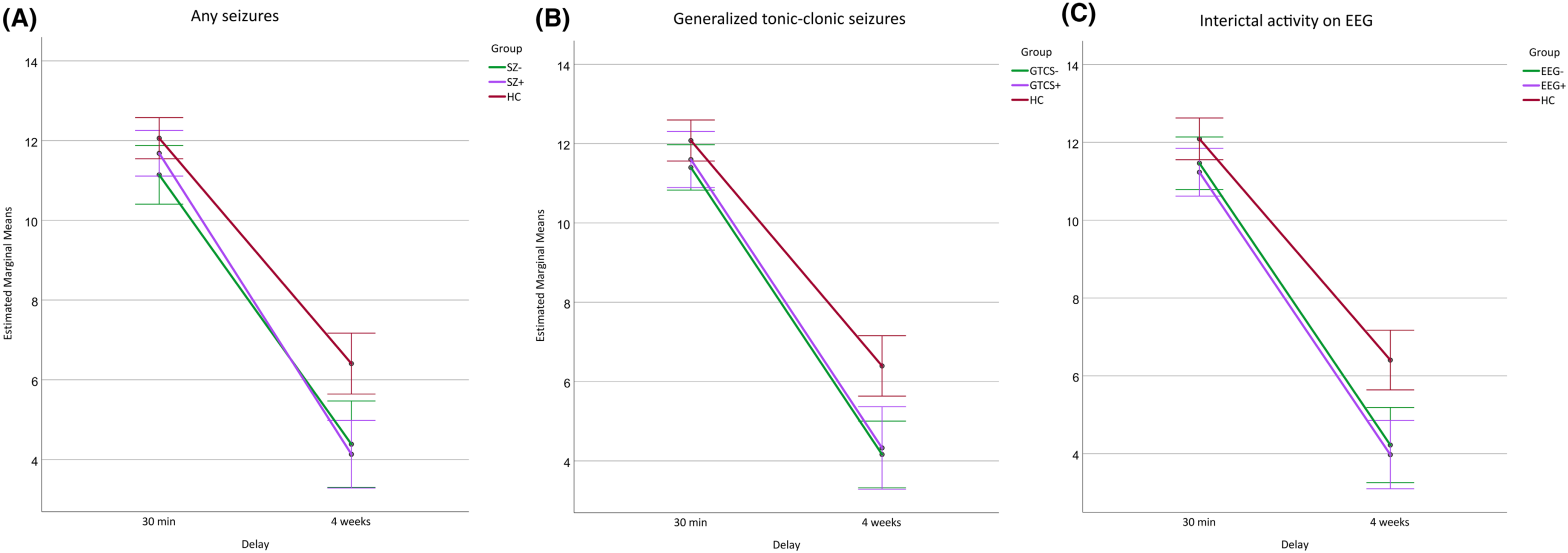
Performance during the Rey Auditory Verbal Learning Test (RAVLT) at 30 min and 4‐week delays after adjustment for the last learning trial in healthy controls (HC) and groups of people with epilepsy subdivided based on (A) any (SZ+/SZ−) or (B) generalized tonic–clonic (GTCS+/GTCS−) seizures experienced within the 4‐week interval between testing occasions, or (C) bilateral (TLE) or generalized (GGE) interictal activity observed before testing (EEG−/EEG+). Estimated marginal means and 95% confidence intervals are presented.

Results of long‐term recall between HCs and PWE grouped by seizure activity are presented in Figure 3. Comparison of performance between PWE groups based on seizures or EEG testing suggested that, after adjustment for the last learning trial, PWE performed similarly to HCs in the RAVLT after 30 min but scored less after 4 weeks irrespective of seizures or bilateral/generalized discharges on the EEG (Table S1, Figure 4). There was no statistically significant group by delay interaction for the VLS task and PWE subgroups were observed to have lower ROCFT scores than HCs at both 30 min and 4‐week delays.

## 4 DISCUSSION

We compared long‐term memory patterns among PWE with TLE and GGE as well as healthy participants in tasks of word, story, and figure recall. Indications of ALF in the task of word recall have been shown previously among patients with GGE as opposed to HCs.^8^ The current analysis of this task suggested that, if the analysis is adjusted for the last learning trial, individuals with R‐TLE perform worse than HCs after delays of both 30 min and 4 weeks. On the other hand, patients with L‐TLE and GGE had a pattern characteristic of ALF – relatively normal performance after 30 min and increased forgetting afterward. Therefore, it may be thought that patients with R‐TLE forget significantly more information than patients with L‐TLE or GGE just after learning and before the first delay. It remains unknown whether such results could emerge because of an earlier (i.e., before the 30 min delay) or greater acceleration of forgetting in R‐TLE or if they represent a qualitative difference of word forgetting patterns among the epilepsy subgroups.^17^, ^18^ It should be acknowledged that the pattern characteristic of ALF in the task of word recall was detected only after adjustment for the result of the last word learning trial. If general performance was considered, PWE demonstrated poorer acquisition of verbal material as well as worse recall at 30 min and 4 weeks. Moreover, the pattern of ALF was not predominant on an individual level and was detected for 6%–23% of patients in the PWE subgroups. These considerations suggest that the detection of a forgetting pattern resembling ALF in our study was (1) highly dependent on statistically equated learning results across subgroups and (2) was probably driven by subtle trends that are not substantial on an individual level, but average to result in an apparent acceleration of forgetting between 30 min and 4 weeks within the GGE and L‐TLE subgroups. After grouping PWE based on ictal events rather than the type of epilepsy, accelerated forgetting was observed between delays of 30 min and 4 weeks both in those patients who presented with seizures and those without. This further suggests that ictal events may not be essential for an increase in the rate of forgetting among PWE, especially considering the growing body of evidence that ALF is not epilepsy‐specific.^5^, ^6^, ^19^ On the other hand, it may be noted that lesional causes of epilepsy (however, rarely including hippocampal sclerosis) were present in most individuals with ALF. Given the limited number of TLE cases in the current study, we could not provide robust evidence of this association, but our data is in line with previous findings, suggesting that the etiology of epilepsy differentially affects ALF and that ALF is rare in patients with hippocampal sclerosis.^20^

To the best of our knowledge, there has been only one previous study simultaneously investigating story and visual recall in TLE and GGE.^6^ Its authors reported accelerated forgetting in TLE during a 3‐week delay of items and descriptive recall of visual scenes as well as story recognition. However, patients with TLE demonstrated normal spatial recall of visual scenes and story recall while those with GGE were unimpaired in all the tasks. Our data support the finding of similar patterns of story recall in HCs and PWE with either GGE or TLE. The visual memory task consisted of the Rey‐Osterrieth figure rather than visual scenes. It indicated that PWE subsamples performed worse after 30 min as well as 4‐week delays irrespective of ictal events. While a progressive forgetting curve of visual material could be expected in patients with TLE based on previous reports, the close performance of subgroups of GGE and TLE in this task opposes the notion that GGE has only a minor impact on long‐term memory function.^9^, ^21^, ^22^

The data presented should be interpreted considering their limitations. These include the fact that only free material recall was tested without investigating recognition and immediate recall of the Rey‐Osterrieth figure was not measured.^13^ Further, initial learning deficits were adjusted by statistical methods rather than by manipulating the exposure to testing material upon learning.^6^ While the impact of initial learning on forgetting curves is not established, our findings should be replicated by matching immediate recall as well as enrolling IQ‐matched controls.^13^ Finally, part of our results may be determined by the characteristics of our PWE sample in which most patients were on antiseizure medication and were not seizure‐free, thus suggesting that the general epilepsy‐related impairment of cognitive functions was possibly higher than in the previous reports.^6^

## 5 CONCLUSIONS

A direct comparison of the recall of verbal material between patients with TLE and GGE suggested different forgetting patterns in these groups. After a short delay, the learning‐adjusted recall was worse in R‐TLE as compared to L‐TLE, GGE, and HCs. Patients with L‐TLE and GGE performed worse than HCs only after the delay of 4 weeks, suggesting ALF in both of these subgroups after adjusting for learning capacity. After grouping PWE based on bilateral or generalized EEG discharges, loss of word list information over time among patients with epilepsy was also consistent with ALF and there were no indications of different forgetting patterns based on seizures or interictal epileptiform discharges. Despite PWE underperforming in the task of verbal story recall, no significant between‐group differences in forgetting curves were noted. Finally, continuously greater forgetting of visual material in both TLE and GGE subsamples was observed. Further trials are required to confirm our findings and better define the differences in forgetting patterns between patients with TLE and GGE.

## FUNDING INFORMATION

This project has received funding from the Research Council of Lithuania (LMTLT), agreement No P‐SV‐22‐33.

## CONFLICT OF INTEREST statement

None of the authors has any conflict of interest to disclose. We confirm that we have read the Journal's position on issues involved in ethical publication and affirm that this report is consistent with those guidelines.

## Supporting information


Table S1.
