# Supplementary material for: Longer‐term verbal and visual memory patterns in patients with temporal lobe and genetic generalized epilepsies
Source: Epilepsia Open. 2023 Aug 10;8(4):1279–87. doi: 10.1002/epi4.12779 (PMC10690666; doi:10.1002/epi4.12779)
Supplement: Supplementary file 1 — Table S1. [file EPI4-8-1279-s001.docx]

|  | PWE subgroups based on epileptic activity | | |
| --- | --- | --- | --- |
| Task | SZ+/SZ- (n=129)^a^ | GTCS+/GTCS- (n=129)^a^ | EEG+/EEG- (n=132) |
| RAVLT |  |  |  |
| Delay | F(1,125)=0.1, p=0.726, $\eta_{p}^{2}$<0.01 | F(1,125)=0.4, p=0.534, $\eta_{p}^{2}$<0.01 | F(1,128)=0.4, p=0.515, $\eta_{p}^{2}$<0.01 |
| Delay by last learning trial | F(1,125)=8.5, p=0.004, $\eta_{p}^{2}$=0.06 | F(1,125)=7.5, p=0.007, $\eta_{p}^{2}$=0.06 | F(1,128)=7.3, p=0.008, $\eta_{p}^{2}$=0.05 |
| Group | F(2,125)=7.3, p=0.001, $\eta_{p}^{2}$=0.10 | F(2,125)=7.3, p=0.001, $\eta_{p}^{2}$=0.10 | F(2,128)=8.6, p<0.001, $\eta_{p}^{2}$=0.12 |
| Delay by group | F(2,125)=4.6, p=0.012, $\eta_{p}^{2}$=0.07 | F(2,125)=3.9, p=0.022, $\eta_{p}^{2}$=0.06 | F(2,128)=4.1, p=0.019, $\eta_{p}^{2}$=0.06 |
| Post hoc (Bonferroni adjusted) |  |  |  |
| RAVLT 30 min | HC=SZ+ (p=1.000), HC=SZ- (p=0.455), SZ-=SZ+ (p=0.753) | HC=GTCS+ (p=0.902), HC=GTCS- (p=0.282), GTCS-=GTCS+ (p=1.000) | HC=EEG+ (p=0.151), HC=EEG- (p=0.481), EEG-=EEG+ (p=1.000) |
| RAVLT 4 weeks | HC>SZ+ (p=0.001), HC>SZ- (p=0.009), SZ-=SZ+ (p=1.000) | HC>GTCS+ (p=0.008), HC>GTCS- (p=0.001), GTCS-=GTCS+ (p=1.000) | HC>EEG+ (p<0.001), HC>EEG- (p=0.002), EEG+=EEG- (p=1.000) |
| VLS |  |  |  |
| Delay | F(1,125)=1.4, p=0.243, $\eta_{p}^{2}$=0.01 | F(1,125)=0.2, p=0.631, $\eta_{p}^{2}$<0.01 | F(1,128)=0.5, p=0.497, $\eta_{p}^{2}$<0.01 |
| Delay by immediate recall | F(1,125)=21.2, p<0.001, $\eta_{p}^{2}$=0.15 | F(1,125)=16.8, p<0.001, $\eta_{p}^{2}$=0.12 | F(1,128)=19.7, p<0.001, $\eta_{p}^{2}$=0.13 |
| Group | F(2,125)=2.6, p=0.078, $\eta_{p}^{2}$=0.04 | F(2,125)=1.0, p=0.375, $\eta_{p}^{2}$=0.02 | F(2,128)=1.4, p=0.244, $\eta_{p}^{2}$=0.02 |
| Delay by group | F(2,125)=2.7, p=0.069, $\eta_{p}^{2}$=0.04 | F(2,125)=0.2, p=0.810, $\eta_{p}^{2}$<0.01 | F(2,128)=1.4, p=0.262, $\eta_{p}^{2}$=0.02 |
| ROCFT |  |  |  |
| Delay | F(1,125)=1.2, p=0.279, $\eta_{p}^{2}$=0.01 | F(1,125)=1.0, p=0.328, $\eta_{p}^{2}$=0.01 | F(1,128)=1.5, p=0.224, $\eta_{p}^{2}$=0.01 |
| Delay by ROCFT-copy | F(1,125)=5.9, p=0.016, $\eta_{p}^{2}$=0.05 | F(1,125)=5.4, p=0.022, $\eta_{p}^{2}$=0.04 | F F(1,128)=6.7, p=0.011, $\eta_{p}^{2}$=0.05 |
| Group | F(1,125)=24.6, p<0.001, $\eta_{p}^{2}$=0.28 | F(2,125)=22.7, p<0.001, $\eta_{p}^{2}$=0.27 | F(2,128)=23.2, p<0.001, $\eta_{p}^{2}$=0.27 |
| Delay by group | F(2,125)=0.7, p=0.483, $\eta_{p}^{2}$=0.01 | F(2,125)=0.1, p=0.920, $\eta_{p}^{2}$<0.01 | F(2,128)=1.6, p=0.203, $\eta_{p}^{2}$=0.03 |

**Supplementary Table 1.** Forgetting patterns in healthy controls (HC) and groups of people with epilepsy (PWE) subdivided based on any (SZ+/SZ-) or generalized tonic-clonic (GTCS+/GTCS-) seizures experienced within the 4-week interval between testing occasions, or bilateral (TLE) or generalized (GGE) interictal activity observed before testing (EEG-/EEG+). RAVLT – The Lithuanian equivalent of the Rey Auditory Verbal Learning Test, VLS – The Verbal-Logical Story Test, ROCFT – The Rey–Osterrieth Complex Figure Test, a – for three individuals with TLE, the presence or absence of seizures during the 4-week delay was unknown.
